# Expression of CD44 and Its Spliced Variants: Innate and Inducible Roles in Nervous Tissue Cells and Their Environment

**DOI:** 10.3390/ijms26178223

**Published:** 2025-08-24

**Authors:** Maria Concetta Geloso, Francesco Ria, Valentina Corvino, Gabriele Di Sante

**Affiliations:** 1 Section of Human Anatomy, Department of Neuroscience, Università Cattolica del Sacro Cuore, Largo Francesco Vito 1, 00168 Rome, Italy; valentina.corvino@unicatt.it; 2 Gemelli Science and Technology Park (GSTeP)-Organoids Research Core Facility, Fondazione Policlinico Universitario Agostino Gemelli IRCCS, 00168 Rome, Italy; 3 Section of General Pathology, Department of Translational Medicine and Surgery, Università Cattolica del Sacro Cuore, Largo Francesco Vito 1, 00168 Rome, Italy; francesco.ria@unicatt.it; 4 Department of Medicine and Surgery, Section of Human, Clinical and Forensic Anatomy, University of Perugia, 06132 Perugia, Italy; gabriele.disante@unipg.it

**Keywords:** CD44 expression in astrocytes, CD44 expression in microglia, CD44 expression in neurons, blood–brain barrier, CD44 variants

## Abstract

CD44, a structurally diverse cell-surface glycoprotein, plays a multifaceted and indispensable role in neural tissue across both physiological and pathological conditions. It orchestrates complex cell–extracellular matrix interactions and intracellular signaling through its variant isoforms and post-translational modifications and is broadly expressed in neural stem/progenitor cells, microglia, astrocytes, and selected neuronal populations. The interactions of CD44 with ligands such as hyaluronan and osteopontin regulate critical cellular functions, including migration, differentiation, inflammation, and synaptic plasticity. In microglia and macrophages, CD44 mediates immune signaling and phagocytic activity, and it is dynamically upregulated in neuroinflammatory diseases, particularly through pathways involving Toll-like receptor 4. CD44 expression in astrocytes is abundant during central nervous system development and in diseases, contributing to glial differentiation, reactive astrogliosis, and scar formation. Though its expression is less prominent in mature neurons, CD44 supports neural plasticity, circuit organization, and injury-induced repair mechanisms. Additionally, its expression at nervous system barriers, such as the blood–brain barrier, underscores its role in regulating vascular permeability during inflammation and ischemia. Collectively, CD44 emerges as a critical integrator of neural cell function and intercellular communication. Although the roles of CD44 in glial cells appear to be similar to those explored in other tissues, the expression of this molecule and its variants on neurons reveals peculiar functions. Elucidating the cell-type-specific roles and regulation of CD44 variants may offer novel therapeutic strategies for diverse neurological disorders.

## 1. Introduction

Cluster of Differentiation (CD)44 is a class I transmembrane cell-surface glycoprotein involved in a variety of cellular functions, including cell adhesion, growth, survival, differentiation, and motility [1]. It also plays an important role in mediating interactions between cells and the surrounding environment by binding to components of the extracellular matrix (ECM), the scaffold in which cells are embedded in any tissue. CD44 protein(s) is (are) composed of an extracellular domain, consisting of a hematopoietic binding link, a transmembrane helix, and a cytoplasmic tail interacting with different adaptor and cytoskeletal proteins.

**Figure 1:**
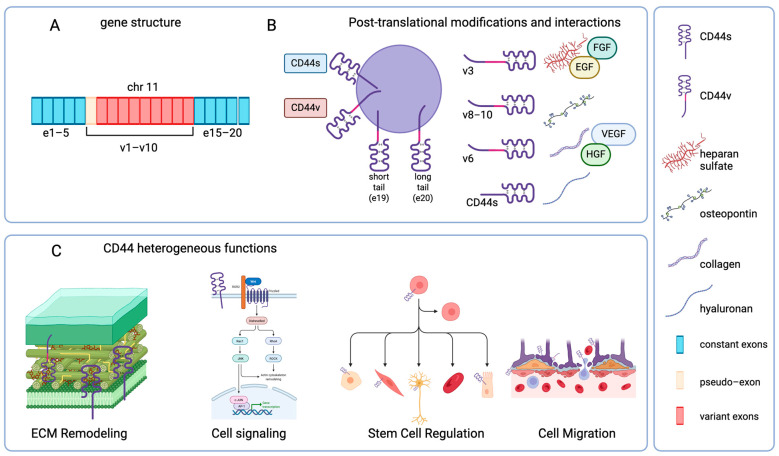
Modular structure and functional diversity of CD44. (**A**) *CD44 gene structure*. The CD44 coding gene and exons are located on human chromosome 11p13. (**B**) Post-translational modifications and interactions. The mature protein is composed of an extracellular domain, a transmembrane helix, and a cytoplasmic tail of two distinct lengths. Post-translational modifications are displayed, and examples of already reported interactions between CD44 isoforms and potential preferential ligands are provided. (**C**) Main functional roles played by CD44. CD44 standard isoform impacts the distribution of molecules of the extracellular matrix (ECM), is involved in different cellular pathways mainly affecting cytoskeletal and proliferative mechanisms and is associated with the modulation of distinct neural and non-neural stem cell lineages. CD44s and other isoforms are involved in the interaction of immune cells with tissue barriers, comprising central nervous system barriers. Created in BioRender. Di sante, G. (2025) https://BioRender.com/5binsw1.

CD44 is encoded by a highly conserved gene located on human chromosome 11p13 and murine chromosome 2. In humans, it spans approximately 60 Kb and comprises 19 exons, whereas in mice, it contains 20 exons [2] (Figure 1A). The structural heterogeneity and the multiple roles played by this glycoprotein derive primarily from the transcriptional regulation and post-transcriptional modifications. CD44 pre-mRNA comprises 10 constant exons (exons e1–e5, e16–e20), a pseudo-exon (v1, non-coding in humans), and 9 variable exons (v2–v10). Alternative splicing can generate 38 potential transcripts (for review, see [3]). The fully spliced transcript (containing only the constant exons) is named CD44 standard (CD44s) and represents the most expressed variant in most unstimulated human cells. The other CD44 variants (CD44v) can contain 1 or a combination of several non-constant exons; their expression is usually tissue-specific and/or inducible in proliferative or pathologic conditions [2,4,5] (Figure 1A).

The fine regulation of alternative splicing and the modulation of CD44s are related to different functions [5]. The alternative splicing of the variant exons encodes for the extracellular portion of the CD44 protein, regarding the part of this molecule able to interact with ECM. In humans, it has been demonstrated that the cytoplasmic tail of CD44 can also undergo a mechanism of alternative splicing: in fact, the last two constant exons can be alternatively selected for encoding, generating a short-tail CD44 (with e19) or a long-tail CD44 (with e20). This phenomenon produces distinct intracellular domains, which impact the signaling pathways [6] (Figure 1B).

Alternative splicing of CD44 is tightly regulated by transacting RNA-binding proteins. Epithelial Splicing Regulatory Protein 1/2 promotes inclusion of v8–10, while heterogeneous nuclear ribonucleoprotein M bolsters the expression of CD44s [7].

It has been demonstrated that glycosylation, glycosaminoglycan addition (such as heparan sulfate on v3), sulfation, and other post-translational modifications further diversify CD44 structure (with a molecular weight range comprised between the 37 to 80 kDa of CD44s to >250 kDa of the largest CD44v), docking conformation, and consequently functions of CD44 [8]. For example, the binding with its main ligands, such as hyaluronan (HA), osteopontin (OPN), and type I collagen, relies on conserved disulfide bridges and secondary structural features, as confirmed through crystallography and deep learning-based protein modeling [4,9,10]. Thus, the modular design of CD44 engenders functional specialization.

Functionally, CD44 mediates basal cell adhesion and ECM protein(s)-dependent signaling. CD44v gain additional binding motives. Thus, v3 enables heparin-binding growth factor capture [Fibroblast Growth Factor and Epidermal Growth Factor (EGF)], while v6 harbors binding sites of Hepatocyte Growth Factor and Vascular Endothelial Growth Factor (VEGF) [11]. The structural diversity of CD44 essentially contributes to its adaptability and flexibility in participating in multiple processes like ECM interactions, wound healing, matrix remodeling, stem cell niche signaling, and many pathological disorders (Figure 1C).

Due to its abundant expression during critical stages of embryonic development, CD44 has been considered a key player in morphogenesis [12] and hematopoiesis [13]. It contributes to the maturation of lymphocytes since the early stages [14], and its expression is modulated during antigen receptor stimulation [15], mitogenic signaling, and immune cell trafficking during inflammation [2,4,16]. A significant body of literature examined the role of CD44 in pathological disorders such as atherosclerosis [17], cardiovascular diseases [18], and tumorigenicity [19], especially in distinct lymphoma subtypes [20]. The complex regulation of alternative splicing of CD44 pre-mRNA into many different variants has been the object of several studies, mostly in tumor cells [21,22].

CD44s is primarily associated with epithelial-to-mesenchymal transition (EMT), a key process in cancer progression where epithelial cells lose polarity and adhesion, acquiring mesenchymal properties that enhance migratory and invasive capabilities. Mechanistically, CD44s facilitates EMT by promoting the activation of Rho family GTPases, such as RhoA and Rac1, which regulate cytoskeletal dynamics essential for motility [22]. In addition, CD44s can activate the phosphoinositide 3-kinase/protein kinase B (PI3K/AKT) signaling cascade, contributing to increased cell survival, proliferation, and resistance. This isoform is also linked to the repression of epithelial markers like E-cadherin and the upregulation of mesenchymal markers such as vimentin and N-cadherin, further reinforcing the mesenchymal phenotype [9,23].

In contrast, CD44v, particularly CD44v6 and CD44v8–10, enable interactions with specific ligands and co-receptors. CD44v6 functions as a co-receptor for tyrosine kinases such as MET and EGFR, facilitating their ligand-induced activation and downstream signaling via the Ras/mitogen-activated protein kinase [24,25] and PI3K/AKT pathways, which promote proliferation, survival, and metastatic potential [26]. CD44v8–10 has been shown to stabilize the cystine/glutamate antiporter xCT (SLC7A11), enhancing intracellular cystine uptake and promoting glutathione synthesis, which is crucial for maintaining redox homeostasis and protecting cancer cells from reactive oxygen species-induced apoptosis [27]. Reports from several groups converge on a relevant role for the β-catenin pathway in regulating CD44 alternative splicing [28,29].

In the nervous system, the modulation of CD44 expression was found to be associated with different neurologic and psychiatric diseases, also involving the impairment of high cognitive functions. For instance, an increased expression of CD44 has been found in the blood and brain of patients with Alzheimer’s disease (AD) [30], and a role of CD44 in amyloid β (Aβ) plaque formation has been reported [31]. A downregulation of CD44 was described in the cerebrospinal fluid (CSF) of patients with depression [32], while genetic studies in suicide victims highlighted that this glycoprotein is a possible risk gene for suicide [33]. Notably, its downregulation in areas deeply involved in cognitive functions, such as Brodmann areas BA24 (anterior cingulate) and BA9 (dorsolateral and medial prefrontal cortex) of suicide attempters, was highlighted [33]. Taken together, these findings suggest that CD44 plays relevant roles in neuronal functions. However, to date, the neurobiological mechanisms through which CD44 acts in the brain have not been fully elucidated. The present review summarizes the current knowledge on CD44 cell-type-specific expression and function in the central and peripheral nervous system (CNS and PNS) to provide a consolidated, up-to-date understanding of the roles of CD44 in neural tissue.

## 2. The Role of CD44 in the Inflammatory Cells of the Central Nervous System

### 2.1. CD44 Expression in Microglia and Brain Macrophages

Microglia and monocytes are both members of the mononuclear phagocyte system and play important functions both in maintaining brain homeostasis and in defense responses [34]. In physiological conditions, parenchymal microglia represent the predominant myeloid subpopulation, followed by border-associated macrophages (BAMs), which include meningeal, perivascular, and choroid plexus macrophages [34,35]. In pathological conditions, microglia and their blood-borne counterparts (BAMs) expand and participate in the defense responses, exerting different, although not yet definitely clarified, roles [34,36]. CD44 is differently expressed by these two subpopulations of cells in different contexts, reflecting the considerable diversity in gene expression detected in these cells [34,36].

In human nervous tissue, under basal conditions, the expression of CD44 was shown to be restricted to the BAM counterpart [37] and, more specifically, to definite subsets of BAMs [38]. In pathological conditions, CD44 has been identified as a reliable marker of CNS immune-related infiltrating cells [39], as happens, e.g., in the experimental autoimmune encephalomyelitis (EAE) [40]. In this model, CD44 is involved in phagocytosis [41], by which BAMs participate in the resolution of the inflammatory response, eliminating infiltrating cells and clearing debris of apoptotic cells [42]. CD44 is also involved in the interaction of macrophages with ECM via its binding with HA. It has been reported that CD44, interacting with HA, modulates macrophage response to lipopolysaccharide (LPS) through the upregulation of A20, a negative regulator of Toll-like receptor 4/Myeloid Differentiation primary response 88/TIR-Domain-Containing Adaptor-inducing interferon β pathway [43].

Once within the CNS, macrophages upregulate CD44 persistently, particularly around ischemic or injury sites, where they contribute to matrix remodeling, cell migration, and modulation of inflammatory signaling. In addition, CD44 expression is related to the ability of macrophages (of the M1 phenotype) to infiltrate glioma through interaction with OPN signaling, suggesting an important role of this glycoprotein also in the dynamics of glioma progression [44,45].

Variant isoforms of CD44 may further refine this process: for example, splice variants like CD44v7–v8 are linked to active lesions in multiple sclerosis (MS) patients, suggesting they enhance targeted adhesion, retention, or specific ligand interactions in inflamed CNS areas, involving several immune cells [4]. In MS, expression of CD44v3, v7, and v10 has been detected in foamy macrophages located in active and pre-active lesions, while in chronically inactive lesions, these cells were found to express CD44v3 [4,46]. Moreover, it has been reported that CD44v6 expression on the infiltrating monocytes is increased in a model of encephalitis in *rhesus macaques* [47] and in murine models of MS [48].

Expression of CD44 has also been described in activated CD11b^+^ microglia both in vitro, upon LPS challenge [49], and in vivo, in experimental models of different neurologic diseases, such as ischemic stroke [50,51], ALS [52], optic nerve crush [53], cerebral cortex injury [53], EAE, AD, frontotemporal dementia [54], aging [38], and spinal cord injury [55]. Collectively, CD44 expression appears to be associated with microglial activation. It is known that, when microglia become activated, they migrate toward the site of damage, dynamically interacting with the ECM. In vitro experiments pointed out that chondroitin sulfate proteoglycan, one of the main components of the ECM and glial scar, directly activates microglia/macrophages via CD44 [55]. Although the molecular pathways regulating microglia/ECM interplay have not been fully elucidated, the interaction between the ECM protein serglycin (SRGN) and CD44 expressed by microglia has been recently described in an experimental model of stroke [51]. The binding of SRGN to CD44 induces activation of the nuclear factor-ĸB p65 signaling pathway and, importantly, mediates the activation of a proinflammatory microglia phenotype, increasing the production and release of proinflammatory molecules [51]. 

### 2.2. CD44 Expression in Astrocytes

Astrocytes play many different homeostatic roles in the healthy nervous tissue, including clearance of ions and neurotransmitters in the extracellular space, trophic and metabolic support to neurons, regulation of blood flow, and drainage of interstitial fluid (for review, see [56]). All these activities require tightly controlled interactions with the ECM, of which they are active effectors of remodeling and structure [56]. ECM, in turn, mediates astrocyte interactions with both neurons and other glial cells. For this reason, it is not surprising that many studies have identified astrocytes as the principal source of CD44 in the brain (reviewed by [57]). CD44 does not seem to be constitutively expressed by all astrocytes, being detectable only in specific subpopulations. In the human brain, CD44 expression identifies a specific astrocyte subset localized in cortical columns, corresponding to intralaminar astrocytes [58]. They are characterized by a soma localized in layer I and long processes that extend radially toward deep cortical layers [59]. Their CD44^+^ long processes [58] cross the domains of protoplasmic astrocytes, thus possibly affecting the functional properties of the astrocytic syncytium by increasing, for instance, its volume and the speed of communication [58]. More recently, in addition to the isocortical localization, similar CD44^+^ astrocytes have been found in many other regions of the human CNS, such as the striatum, thalamus, brainstem, cerebellum, and spinal cord, besides being diffusely present in the white matter, subependymal, and subpial regions [60].

Astrocytes originate from neural precursor cells, and their differentiation process is associated with expression of numerous ECM molecules, such as proteoglycans and tenascins [61], as well as related receptors, among which is CD44 [62,63,64,65,66]. Its expression even characterizes a specific astrocyte-restricted precursor cell subpopulation [64]. The amount of CD44 decreases during development, as shown in the developing mouse cerebellum, where CD44, expressed by astrocyte precursors located in the white matter and by Bergman glia, is undetectable in adult life [65,67]. Accordingly, in the human telencephalon, CD44 shows the highest expression in astrocytes during the fetal and neonatal periods [58]. In line with this observation, prenatal expression of CD44 has also been detected in a subpopulation of retinal neuroglia, represented by Müller cells [68], which share many functional and structural features with astrocytes [69].

**Figure 2:**
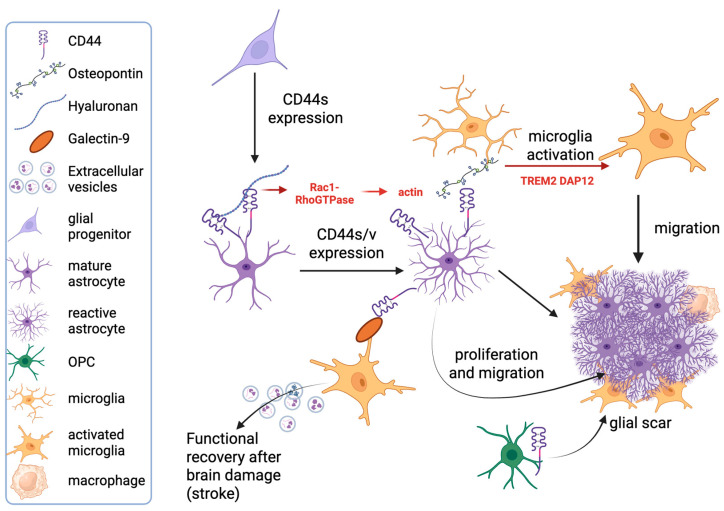
CD44 expression in glial cells. Diagram representing different roles played by CD44 expression in different glial lineages. The main molecular pathways involved are schematically indicated. As shown, CD44 participates in the differentiation process of astrocytes from progenitors, plays a role in both astroglia and microglia activation, and is involved in the tissue reaction to damage. Dap12: DNAX Activation Protein of 12 kDa; OPC: oligodendrocyte precursor cell; TREM2: Triggering Receptor Expressed on Myeloid cells 2. Created in BioRender. Di sante, G. (2025) https://BioRender.com/5binsw1.

Most of the current literature on the expression of CD44 in astrocytes points to its association with pathological conditions. A marked upregulation of CD44 expression has been detected in tumors originating from astrocytes (gliomas) and oligodendrocytes (oligodendrogliomas), in line with the widely described expression of CD44 in neoplasms [70,71]. However, its description lies beyond the scope of this review. Instead, we will focus on CD44 upregulation related to reactive astrogliosis, that is, the astrocyte reaction to pathological conditions affecting the nervous system [56] (Figure 2). In both animal models and human tissue, increased expression of CD44 in reactive astrocytes has been found in a wide range of neurologic pathologies, including hypoxia/ischemia [60,72], epilepsy [60,73,74,75,76], MS and related experimental models [77,78,79,80], prion disease [81], tuberous sclerosis [82], AD [30,83,84,85], and other neurodegenerative conditions such as Parkinson’s disease (PD) [86] and ALS [52]. In line with the above-mentioned results, the deletion of CD44 has been shown to reduce reactive astrogliosis and improve clinical features [73]. On the other hand, conflicting results have also been reported because, during prion disease, CD44 deletion was unable to impair both astrocyte and microglial activation, as well as the development of prion disease pathology [81].

Much evidence suggests that CD44 provides mechanistic contributions to the complex cascade of events that characterize reactive astrocytosis. One is represented by its role in the marked morphological changes that are a typical feature of reactive astrocytes [87]. Indeed, depletion of CD44, as well as degradation of HA, induces Rac1 [88], whose activation influences astrocyte morphology, acting at the level of the actin cytoskeleton [89]. Moreover, Rac1 activation, together with protein kinase (PK)Nγ signaling, also regulates cell migration [90], which is another feature of reactive astrocytes [91].

There are different subtypes of reactive astrocytes [87], and, in this regard, transcriptomic studies highlighted that CD44 is part of the transcriptional profile of some of these specific subsets in many different neurodegenerative conditions [60,87,92,93,94,95,96,97].

The transcriptional profile of the different CD44^+^ astrocyte subtypes appears highly dependent on the nature of the inducing stimulus [93]. In this respect, proteins involved in ECM structure, as well as adhesion molecules, among which CD44, emerge as one of the largest classes of genes deregulated in these reactive astrocytes, even though the extent to which any gene is induced depends on the stimulus [93]. In particular, collagen, versican, a large extracellular matrix proteoglycan, and CD44 are more expressed in contexts showing overt neuronal damage and consequent formation of glial scar, such as ischemic stroke, compared with transient neuroinflammation induced by peripheral challenge [93,98]. CD44 plays a relevant role in fibrotic scar formation not only when expressed by local fibroblasts [94], responsible for tissue remodeling and fibrosis [95], but also when expressed by astrocytes (Figure 2), which form the outer border of the scar and are involved in reducing the spread of damage [95]. Indeed, its interaction with the high-molecular-weight (HMW) form of HA, which is directly produced by reactive astrocytes in pathological conditions, such as EAE [96], induces contact inhibition of astrocytic growth and proliferation [99] (Figure 2).

An important mechanism through which CD44 expressed by reactive astrocytes participates in the tissue reaction to damage is its interaction with OPN [97]. In a rodent model of stroke, CD44 expression was found in both reactive astrocytes and proliferating oligodendrocyte precursor cells (OPCs) located at the lesional rim, close to OPN^+^ myeloid cells [72]. OPN is also expressed by specific subsets of microglia, as indicated by single-cell RNA-seq performed in both MS and related experimental models [100], thus allowing us to hypothesize a role of the CD44-OPN axis in facilitating the crosstalk among different types of glial cells. Recent reports in a model of AD show that, in the peri-amyloid plaque environment, the astrocytic signals mediated by CD44, Clusterin, and ApoE target the Triggering Receptor Expressed on Myeloid (TREM)2 signaling pathway in microglia by binding to TREM2 and its downstream partner DNAX Activation Protein of 12 kDa (Dap12), inducing microglia activation [101]. The interaction between the immunomodulator protein galectin-9 (LGALS9) and CD44 is a novel pathway emerging as a mediator of the crosstalk between CD44^+^ astrocytes and activated microglia expressing LGALS9 [98]. Interestingly, this interaction is associated with neuroprotective effects, enhancing mice recovery from stroke and promoting remyelination [98]. Together, these data point to CD44 as a central player in the modulation of cell–cell interactions in nervous tissue reaction to damage (Figure 2).

Regarding the alternative splicing of this adhesion molecule, although CD44s is strongly positive and widely distributed in the glial matrix, it is weakly expressed on glial cells [102]. CD44v6, known to be expressed by activated lymphocytes and by metastatic variants of tumor cells, has been identified in vitro in astrocytes activated upon stimulation with inflammatory cytokines [103].

## 3. CD44 Coregulates Myelination of Central and Peripheral Nervous Systems

### 3.1. CD44 and Oligodendrocytes

Oligodendrocytes are nervous tissue cells of crucial importance for proper neuronal functions, since they are responsible for the production of myelin, the multilayered lipidic membrane wrapping axons, which allows the effective propagation of action potentials [104]. OPCs originate from neural stem cells of the subventricular zone, from which they migrate to different regions of CNS white matter and postnatally differentiate into mature oligodendrocytes [104].

Although not numerous, some studies describe CD44 expression in oligodendrocytes. Besides the increased expression reported in tumors derived from the oligodendrocytic lineage [70,105], the expression of CD44 in the CNS appears to be more evident during OPC differentiation [63,70]. In this regard, the finding that CD44 overexpression (in particular CD44s) in oligodendrocytes drives HA accumulation and defects in myelination [106] allows us to hypothesize a possible role for CD44 in the functional activities of this cell type. Indeed, it is known that ECM proteins, among which HA, play a relevant role in the myelination process [104]. Moreover, CD44^+^ oligodendrocytes have been described in adrenoleukodystrophy, in which it has been hypothesized that there is a cytotoxic effect mediated by CD44 through the hyaluronate matrix [107].

However, the role played by the CD44-HA axis in CNS myelination is controversial. There are studies in demyelinating diseases, such as MS [96] and vanishing white matter disease [108], in which the involvement of CD44 in myelination was not evident, despite the finding that astrocytic-induced accumulation of HMW-HA results in defects of OPC maturation and impairment of myelination [96,108]. In addition, CD44 blocking antibodies failed to reverse the HA-induced myelin impairment [109]. On the other hand, more recently, an in vitro study pointed out that HA and CD44 are directly involved in inhibiting morphological differentiation in FBD-102b cells, an in vitro differentiation model of OPCs, and in primary OPCs, an effect mediated by transmembrane protein 2 (TMEM2), a cell surface hyaluronidase [110]. Notably, although the role played by CD44 in myelination remains to be further clarified, other studies suggest that this molecule may affect other activities of this cell type in pathological conditions. Specifically, by analogy with astrocytes, findings suggest the involvement of CD44 in OPCs migration towards the lesion site, as pointed out in a model of demyelination [111] and in a model of stroke [72].

### 3.2. CD44 and Schwann Cells

CD44 has also been detected in the myelinating cells of the PNS, the Schwann cells. Its expression was shown to be particularly marked during their proliferation and maturation phase [112]. In adult life, CD44 is expressed in terminal Schwann cells located at the neuromuscular junction (NMJ) and in the non-myelinating Schwann cell subpopulation [113]. It appears functionally associated with ErbB2 and ErbB3, the neuregulin receptors, whose signaling is important for many functional activities of Schwann cells, including their correct adhesion to neurites [112]. Their localization has also been found at the level of the NMJ, where, in the SOD1 model of ALS, the remodeling that follows axonal degeneration and consequent denervation is associated with increased expression of both CD44 and ErbB3 in terminal Schwann cells, supporting a role of CD44 in synaptic plasticity [113]. In addition, CD44 plays a remarkable role in peripheral nerve regeneration after injury, a process that is strongly dependent on the tightly regulated interaction of Schwann cells with the ECM [114]. In particular, CD44, interacting with HA, controls Schwann cell motility [115], further confirming its role in cell migration. Moreover, it also supports Schwann cell survival upon binding with OPN [116]. Indeed, after peripheral nerve injury, Schwann cells upregulate *Spp1*, encoding OPN, and *Cd44*, promoting Schwann cell proliferation and inhibiting their apoptosis through PKCα signaling, thus improving peripheral nerve recovery [116].

## 4. The Role of CD44 in Neuronal Functions

**Figure 3:**
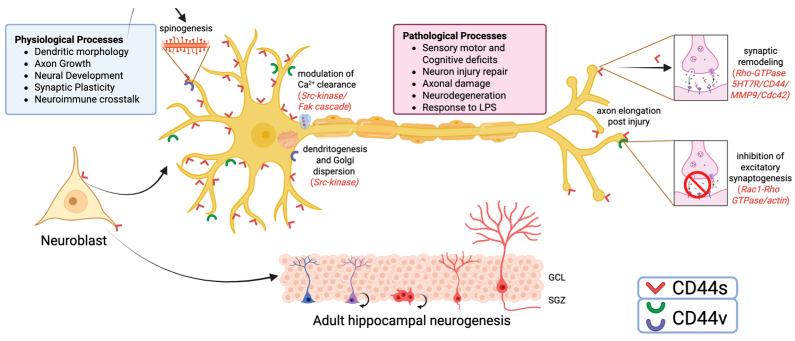
CD44 expression in neurons. Diagram showing different roles played by CD44 in neurons in both physiologic (light blue square) and pathologic conditions (pink square). The main molecular pathways involved are schematically indicated. SRC: steroid receptor coactivator; FAK: focal adhesion kinase; 5HT7R: 5-hydroxy-tryptamine receptor 7; MMP9: metalloproteinase 9; Cdc42: Cell Division Control protein 42; GCL: granular cell layer; SGZ: sub-granular zone. Created in BioRender. Di sante, G. (2025) https://BioRender.com/5binsw1.

Although many studies pointed out that CD44 expression in the nervous tissue is essentially limited to glial cells [57], mounting evidence suggests that it is also present in neurons of both CNS [117] and PNS [118,119]. In basal conditions, in the adult CNS, the presence of CD44 in neuronal cells has been reported, although its expression levels were lower than those found in the developing brain [120]. In particular, CD44 was found in facial motor neurons [121], cerebellar granules [67], and dopaminergic neurons of the rat *substantia nigra* [122] and its splicing variants were observed in different neuronal subpopulations located in selected gray matter regions of the human CNS [102]. A regional selectivity was also confirmed by the expression pattern of CD44 mRNA, which appeared to be prevalently expressed in some limbic regions, such as midline thalamic nuclei, striatum, amygdala, hypothalamus, CA3 region of ventral hippocampus, and layer 6 of several cortical areas [117]. Since most of these areas show involvement in cognition and behavior, a possible role of CD44 in cognitive functions has been hypothesized. Indeed, this hypothesis was corroborated by the finding that CD44 knockout mice exhibit sensory-motor and cognitive deficits, which could be the consequence of neurodevelopmental alterations consequent to the lack of CD44 proteins [123] (Figure 3).

### 4.1. CD44 and Neural Development

The involvement of CD44 in neuronal development and circuit organization emerges from many studies. Its expression was found in immature neurons, such as embryonic optic chiasm neurons [124], cerebellar neuronal precursors (both Purkinje and granule cells) [67], and developing cortical neurons [125]. In this regard, a role in axon growth has also been described in the developing visual system [126] and, in vitro, in SH-SY5Y cells [127]. In the adult rodent brain, CD44 expression has been detected in SOX2+ neural stem cells (NSCs) located in the subgranular zone of the dentate gyrus, one of the brain regions involved in adult neurogenesis [128]. Importantly, it has been shown that CD44 regulates some functional properties of NSCs, such as quiescence and differentiation, since the digestion of HA or genetic ablation of CD44 results in the loss of NSC quiescence [128]. Due to the role played by adult hippocampal neurogenesis in learning and memory [129], this finding represents further evidence supporting the involvement of this molecule in cognition. Mechanistically, the study by Sun and coworkers also demonstrates that the modulation of NSCs’ properties takes place through CD44 binding with HA [130], which, in turn, is known to contribute to cognitive functions and synaptic plasticity through several mechanisms [131] (Figure 3).

Skupien and colleagues showed that CD44 is implicated in dendrite development and morphology. They report that the downregulation of CD44 at the peak of dendritogenesis increases the complexity of dendritic tree morphology, suggesting that this molecule may function as a growth-inhibitory mechanism important in proper neuronal connectivity [120]. CD44-mediated control of dendritogenesis was associated with dispersion of the Golgi apparatus [120], which is known to be involved in both the establishment and maintenance of neuronal polarity [132], as well as in maintaining adequate development of the dendritic tree [133]. Modulation of Golgi morphology occurs through a steroid receptor coactivator (Src)-dependent mechanism, in which Src-kinase acts as a downstream effector of CD44 [120]. Interestingly, in cortical pyramidal neurons, loss of CD44 in vivo differently influenced the branching of apical and basal dendrites [120], which are known to receive different inputs and play different roles in cortical connectivity [134] (Figure 3). In cortical spheroids, CD44 was shown to localize in newly formed excitatory synapses, where it induces an attenuation of Rac1 activity, resulting in a suppression of excitatory synaptogenesis [135]. This is in line with the well-known functional mechanisms of the intracytoplasmic portion of CD44, which, through the ERM-protein link with the actin cytoskeleton, modulates cell morphology via Rho-GTPases as a response to environmental changes [136].

CD44, together with HA synthases 1 and 2, is also expressed by neural crest cells during development, where it regulates their migratory activity [137]. In adult PNS sensory neurons, CD44 is involved in signaling pathways that influence their excitability by controlling cytoplasmic Ca^2+^ clearance, which is under the control of plasma membrane Ca^2+^ ATPase (PMCA). CD44 expressed by sensory peripheral neurons activates the Src family of tyrosine kinases—focal adhesion kinase signaling cascades, which exerts tonic inhibitory activity on PMCA [118]. Functionally, in the PNS, CD44 expressed by nociceptors was shown to modulate pain transmission. In particular, it has been shown that derivatives of HA, such as low molecular weight (LMW)-HA and HMW-HA, act through their binding with CD44 to cause, respectively, hyperalgesia and anti-hyperalgesia, thus suggesting that this receptor could represent a therapeutic target to alleviate inflammatory and neuropathic pain [138].

### 4.2. CD44 in Synaptogenesis and Synaptic Plasticity

In adult neuronal cells, dendrites have been identified as the major site of CD44 structural localization [120,139]. The molecule is expressed at both presynaptic and postsynaptic levels, where it plays a crucial role in the regulation of activity-dependent dendritic spine structural plasticity. Also in this case, CD44 modulates the activity of small Rho-GTPases (RhoA, Rac1, and Cdc42) [139].

Another mechanism involved implies CD44 interaction with the serotonin receptor 5-hydroxy-tryptamine receptor 7 (5-HT7R) that plays a significant role in learning and memory processing [140]. In particular, upon 5-HT7R stimulation, CD44 expressed by dendritic spines is cleaved by activated matrix metalloproteinase (MMP)-9, detaches from ECM, and eventually engages the small Rho-GTPase Cdc42, with the final result of neuronal outgrowth and elongation of dendritic spines that contributes to the expression of long-term potentiation (LTP) [141]. More recently, the same group highlighted that CD44 modulates both the constitutive activity of 5-HT7R and its agonist-mediated signaling. Heteromerization also results in the transactivation of 5-HT7R-mediated signaling via the CD44 ligand [142]. Interestingly, they also noted the developmentally regulated expression of the 5-HT7R-CD44 heteromers in cortical areas involved in high cognitive functions, such as the hippocampus and prefrontal cortex. Based on this mechanism, even small variations in the amount of HA may have a relevant impact on 5-HT7R-mediated signaling [142] (Figure 3).

CD44 also indirectly affects brain plasticity and synaptic functions, through its expression in astrocytes. Astrocytes are an integral component of the synapse, and also reactive astrocytes can contribute to synapse remodeling and circuit reorganization [56,143]. In particular, astrocytes contact synapses with thin peripheral processes known as leaflets, participating in the control of the homeostasis of the synaptic cleft [56,143]. Depletion of CD44 in astrocytes has been shown to impair the interaction of astrocytic leaflets with the synaptic cleft, leading to an increased density of dendritic spines and a decreased size of postsynaptic densities, as shown in a model of temporal lobe epilepsy [73].

### 4.3. The Role of CD44 in Neurons in Pathological Conditions

By analogy with evidence reporting the glial expression of CD44 in a variety of neurologic diseases, the neuronal expression of CD44 was also observed in pathological conditions. For instance, axotomy is associated with CD44 expression in the soma of related neurons, as demonstrated after transection of the facial nerve, which leads to a strong but transient increase in CD44 immunoreactivity in both neuronal cell bodies and regenerating axons [121]. Consistently, the same group showed that transection of the hypoglossal and *vagus* nerves leads to CD44 expression on related motor neurons, while injury of the sensory component of the sciatic nerve was found associated with increased CD44 expression in the *substantia gelatinosa* and Clarke’s column of the affected spinal cord segment [53]. In cultured hippocampal neurons, CD44, through its ligand OPN and β1-integrin signaling, plays a role in the axon elongation/sprouting phase following neuronal injury [144]. In a model of traumatic brain injury, CD44 was found to be upregulated in cortical neurons in association with the upregulation of the hyaluronidase TMEM2. Interestingly, TMEM2 has been shown to produce low molecular weight (LMW)-HA from HMW-HA, which can enter the cell through CD44 and improve endoplasmic reticulum stress, thus protecting the cell from damage [145]. Indeed, upregulation of CD44 and TMEM2 was associated with a neuroprotective effect on secondary neuronal death [146]. Moreover, in a model of sciatic nerve injury, HMW-HA administration, through its binding with CD44, is associated with neuroprotective effects, such as reduction in proinflammatory mediators [147]. Taken together, these results strongly suggest the involvement of CD44 in repair phenomena occurring in the damaged nervous tissue.

There is also evidence suggesting a role for this molecule in the interactions between neurons and immune cells. Indeed, systemic immune challenge induced by LPS administration was able to increase CD44 expression in neurons located in brain areas that do not express such a molecule in physiological conditions, such as in the *area postrema* and motor nucleus of the *vagus* nerve, both involved in the inflammatory reflex, which plays an important role in transmitting to the CNS inflammatory signals arising in the periphery [148,149].

### 4.4. CD44 Variants in Alzheimer’s Disease

A different arrangement of CD44 isoforms is associated with neurologic diseases, such as AD. While in physiological conditions human isoforms CD44v4, CD44v5, and CD44v10 have been described in neurons [102], in AD brains the upregulation of neuronal CD44 and a distinct pattern of CD44v have been shown [30]. By immunohistochemistry, CD44s was detected in neuritic plaques and astrocytes, whereas human variants CD44v3, CD44v6, and CD44v10 were found overexpressed in hippocampal neurons [30]. The same study highlights that CD44v6 and CD44v10 are induced in primary neuronal cultures and neuroblastoma cells upon stimulation with Aβ-peptide. Notably, the inhibition of CD44v10 function protected neurons from Aβ-peptide-induced neuronal death, thus suggesting a direct role of CD44v10 in neuronal death [25] (Figure 3). The molecular pathway underlying this effect is not known, but due to the documented interaction of CD44v10 and the Ephrin Type-A Receptor 2 signaling pathway [150], which plays a role in AD pathogenesis [151], a possible link has been hypothesized [30].

Taken together, this evidence highlights the relevance of CD44 in neuron activity in both physiologic and pathologic processes (Figure 3).

## 5. CD44 in Central Nervous System Barriers

Recent evidence demonstrated that the anatomical idea of the static meningeal three layers should be abandoned in favor of a dynamic idea of the nervous system compartmentalization [152]. The dogma of the CNS as an immune sanctuary should be revised in light of recent demonstrations of the presence of a 4th “meningeal” layer that has been defined as subarachnoid lymphatic-like membrane (SLYM) and could be considered a mesothelium able to subdivide the subarachnoid space into two different compartments [152]. SLYM has been hypothesized as a “diffused cerebral lymph node”, the residency of myeloid and other immune cells, representing an innate immune niche for the surveillance of CSF, or, categorized from other points of view, as a “hyperosmotically detached reticular/inner arachnoid” [153]. Another aspect to be considered is that the CNS contains another type of barrier that seems to be very similar to the BBB but with different anatomical and physiological features. This barrier is the blood–CSF barrier (BCB), which is more permeable than the BBB, is aimed at the balance between blood and CSF, and is more involved in immune surveillance and the regulation of immune cell trafficking into the ventricles [154].

In this context, CD44s and CD44v are expressed by endothelial and perivascular cells, enabling binding to HA. In pathological conditions, increases in HA in the CSF are related to BBB permeability, implying that the CD44-HA interaction destabilizes junctional integrity [155,156]. It remains unclear which CD44 isoforms generate soluble species and whether variant-specific arrangement contributes causally to barrier failure. Specifically, the binding of HA to CD44s expressed by brain endothelial cells reduces transepithelial electrical resistance (TEER), disrupts tight junction organization, and increases barrier permeability, suggesting that the modulation of the tightness of the barrier is CD44-dependent. At the molecular level, this HA/CD44 engagement, facilitated by the aforementioned involvement of small GTPases such as RhoA and Rac1, triggers mechano-transduction that impairs barrier integrity, as evidenced by decreased TEER and increased permeability [155]. Furthermore, CD44 is a hypoxia-responsive gene, and both endothelial and astrocytic cells upregulate CD44 under ischemic stress, amplifying HA/CD44 signaling during neuroinflammation, stroke, or trauma [155].

Mechanistically, HA engagement of CD44s on brain endothelium has been shown to activate downstream signaling that increases permeability (in vitro), and CD44 genetic deficiency alters disease severity and immune infiltration in animal models, indicating CD44s both mediate and modify inflammatory barrier breakdown [155,157].

In addition, the contribution of MMPs in the cleavage of ECM components cooperates with the function of CD44, reinforcing the idea of a coordinated mechanism of barrier disruption through ECM degradation [156].

While CD44s plays a central role in BBB/BCB regulation, variant isoforms and additional adhesion molecules also contribute significantly. CD44 splice variants can associate with integrins (e.g., α3β1), tetraspanins, and epithelial cell adhesion molecules to form membrane complexes located in glycolipid-enriched domains, affecting cell adhesion and signaling [158,159]. Although the expression patterns and specific functions of CD44v isoforms at brain endothelial and choroid plexus barriers are incompletely mapped, lessons from other tissues suggest that these complexes modulate transmigration and retention during inflammation. Beyond CD44, other classic adhesion molecules, including selectins, integrins (lymphocyte function-associated antigen 1/intercellular adhesion molecule 1 and very late antigen 4/vascular cell adhesion molecule 1), and junctional adhesion molecules orchestrate leukocyte rolling, firm adhesion, and diapedesis [160,161]. Regarding the BCB, the expression of CD44 on ependymal cells remains largely unexplored. Its known functions in HA-mediated adhesion, migration, and mechano-transduction suggest that CD44s or variant isoforms may influence ependymal architecture, response to injury, or CSF–parenchyma interactions.

In scenarios where CD44v(s)-driven permeability is elevated, these molecules may compensate or cooperate, highlighting a multifaceted adhesion network, also dependent on different potential ligands, ensuring regulated immune cell trafficking across CNS barriers.

## 6. Therapeutic Perspectives

The vast landscape of roles for CD44 in the CNS summarized just above makes it a suggestive target for therapies. Yet, inhibition of CD44 and, especially, variant-directed approaches have clear precedent and mechanistic rationale but also important safety and translation challenges. Targeting CD44v (notably CD44v6 and CD44v8–10) has been pursued in oncology because these isoforms contribute to growth-factor co-receptor functions and antioxidant defense via stabilization of the xCT (SLC7A11) cystine/glutamate antiporter. Inhibition of xCT or disruption of the CD44v–xCT interaction depletes CD44v-expressing tumor cells and sensitizes them to oxidative stress, suggesting that CD44v-directed therapies could modulate redox-sensitive pathology [27,162]. Clinical efforts with anti-CD44v6 antibody–drug conjugates (e.g., bivatuzumab-mertansine) proved that variant-selective antibodies can deliver potent effects but also produced on-target toxicity. More broadly, anti-CD44 antibodies show anti-inflammatory activity in murine immune models, indicating potential to dampen neuroinflammation if CNS delivery and isoform selectivity are achieved [163]. In the CD44v–xCT axis specifically, small-molecule xCT inhibitors such as sulfasalazine (and other xCT inhibitors) have been used in preclinical studies to selectively affect CD44v^high^ cells, offering a druggable route that could be repurposed or refined for neuroinflammatory or neurodegenerative disorders [164,165]. However, CNS penetration, target engagement in barrier cells, and safety would all need careful evaluation.

Enhancement or functional modulation of CD44s (or selective agonism of beneficial CD44–ligand axes) is less developed but is conceptually plausible for repair and neuroprotection. CD44–HA signaling can influence BBB tightness and mechano-transduction; therefore, blocking pathological HA–CD44 interactions might reduce barrier leakiness, whereas promoting beneficial CD44-mediated repair functions (e.g., supporting stem-cell niche quiescence or aiding clearance/repair by macrophages) would require isoform- and context-specific approaches to avoid unwanted inflammation or tumorigenic effects [155]. Nanobodies and engineered small antibody fragments directed at CD44 (or carrying HA-mimetic payloads) are an attractive technology because of their smaller size, improved tissue penetration, and the possibility of CNS delivery strategies; initial work on CD44 nanobodies and dual-targeted nanobody delivery platforms supports feasibility, but no CNS-targeted CD44 nanobody therapy has yet reached clinical testing [9,166].

In conclusion, CD44 and its variants represent a promising target for a wide range of diseases of the CNS, including (auto)inflammatory diseases, cancer, and neurodegenerative diseases. However, toxicity and selectivity still hamper such a venue. Adding more knowledge on its various roles and producing new and more selective tools is a new route for therapy.

## 7. Conclusions—A Panoramic View of CD44 Roles in the Nervous System

The comprehensive analysis of cell-specific CD44 (and related variants) expression in neural tissue cells underscores its expression in all cell types and its involvement across various physiological and pathological states of the nervous system. Collectively, the main information that emerges is that CD44 expression in different cell types is mainly a consequence of their dynamic interactions with the local microenvironment. For instance, it is involved in developmental processes and glial lineage specification in both the CNS and PNS [62,63,64,65,66,67,68,69,70,106,107,108,109,110,112,124,125,126,127,128,129,130,131], which contributes to translating external messages into changes in intracellular complex signaling pathways, especially by regulating cell–ECM interactions. Its binding with HA and other ECM proteins, acting on the actin cytoskeleton of neural tissue cells, allows the CD44-mediated control of cell migration, as observed in different progenitor cells, as well as in glial cells [72,90,91,111,115] and in morphological changes underlying astroglia activation [76].

The reorganization of the actin cytoskeleton associated with activation of intracellular signaling pathways also represents the mechanistic substrate underlying its role in neuron-specific functions, such as axon growth [126,127], control of dendritogenesis and dendritic morphology, and circuit organization [120,135,139]. Of note is the involvement of CD44 in serotonin-mediated signaling through its receptor 5-HT7R [141,142]. The latter is involved not only in the modulation of brain plasticity and connectivity but also in many neurological and psychiatric conditions, potentially pointing to the 5-HT7R-CD44 axis as a possible therapeutic target [142,167,168]. A gap of knowledge is represented by variant-specific interactions and functions in neurons. We may speculate that they might enable localized synaptic signaling. Moreover, splice variants like CD44v3/v6 might also participate in growth factor signaling modulation, as shown in non-nervous stem cell niches [8].

Another key feature emerging from this analysis is represented by its multifaceted participation in nervous tissue reaction to damage. CD44 acts as a molecular hub in neuroinflammatory responses, modulating both glial activation and immune cell recruitment [39,40]. Functionally, it participates in phagocytosis and ECM interaction, contributing to resolution or propagation of inflammation depending on context [41,43]. Variants, including CD44v6 and CD44v7–v8, are particularly enriched in activated microglia and macrophages at lesion sites, where they facilitate matrix remodeling, migration, and cytokine signaling [4,44,45,47,48,72]. CD44 in astrocytes, especially in its upregulated, reactive form, is central to lesion compartmentalization and reaction to damage [60,73,77,83,86,88,91,169]. Its cooperation with OPN and influence on microglial activation patterns further illustrate a coordinated CD44-driven inflammatory network [72,101].

At the BBB and BCB, CD44 expression on different cell types regulates barrier properties through HA binding and downstream cytoskeletal signaling [4,155,156,170]. Under ischemic stress or inflammatory challenge, CD44 engagement can alter tight junction integrity, increasing paracellular permeability and facilitating leukocyte transmigration [4,50,155]. While the role of CD44s is well documented in these contexts, variant isoforms remain largely unexplored. Based on findings from non-CNS tissues, CD44v6 may enhance co-receptor signaling for VEGFR-2 or c-Met, potentially influencing angiogenic responses at the BBB [21,171,172]. Similarly, CD44v8–10 could stabilize the xCT antiporter, protecting barrier cells against oxidative stress during neuroinflammation [165,173], though this remains hypothetical. Moreover, in the nervous system, this interaction could be important in the context of neurodegenerative diseases, due to the relevance of the xCT-ferroptosis axis in their pathogenesis [174]. However, the absence of isoform-specific barrier expression maps represents a major limitation in understanding how CD44 diversity shapes CNS barrier function.

In conclusion, understanding the intricate mechanisms by which CD44 mediates cell–cell and cell-ECM interactions, and how its signaling pathways are modulated in different neural cell types, offers promising avenues for therapeutic interventions targeting a wide range of neurological disorders. Future research focusing on the precise regulation of CD44 splice variants and their specific ligand interactions in distinct pathological contexts will be crucial for unlocking its full therapeutic potential.

## Data Availability

Not applicable.

## Abbreviations

AD	Alzheimer’s disease
Aβ	Amyloid beta
ALS	Amyotrophic lateral sclerosis
BAM	Border-associated macrophage
BBB	Blood–brain barrier
BCB	Blood–cerebrospinal fluid barrier
CD	Cluster of differentiation
CNS	Central nervous system
CSF	Cerebrospinal fluid
Dap12	DNAX activation protein of 12 kDa
LGALS9	Galectin-9
HA	Hyaluronan
HMW	High molecular weight
HT7R	Hydroxytryptamine Receptor 7
EAE	Experimental autoimmune encephalomyelitis
ECM	Extracellular matrix
EGF	Epidermal growth factor
EMT	Epithelial-to-mesenchymal transition
EAAT	Excitatory amino-acid transporter
LPS	Lipopolysaccharide
LTP	Long-term potentiation
MMP	Matrix metalloproteinase
MS	Multiple sclerosis
NMJ	Neuromuscular junction
NSC	Neural stem cell
OPN	Osteopontin
OPC	Oligodendrocyte progenitor cell
PD	Parkinson’s disease
PI3K/AKT	Phosphoinositide 3-kinase/protein kinase B
PK	Protein kinase
PMCA	Plasma membrane Ca^2+^ ATPase
PNS	Peripheral nervous system
RNA-seq	RNA-sequencing
SLC7A11	Cystine/glutamate antiporter xCT
SRGN	Serglycin
SLYM	Subarachnoid lymphatic-like membrane
TEER	Transepithelial electrical resistance
TLR4	Toll-like receptor 4
TREM	Triggering receptor expressed on myeloid
VEGF	Vascular endothelial growth factor
